# Amylin Uncovered: A Review on the Polypeptide Responsible for Type II Diabetes

**DOI:** 10.1155/2013/826706

**Published:** 2013-03-31

**Authors:** Karen Pillay, Patrick Govender

**Affiliations:** Department of Biochemistry, School of Life Sciences, University of KwaZulu Natal, Westville Campus, Block F3, University Road, Private Bag X54001, Durban 4000, South Africa

## Abstract

Amylin is primarily responsible for classifying type II diabetes as an amyloid (protein misfolding) disease as it has great potential to aggregate into toxic nanoparticles, thereby resulting in loss of pancreatic *β*-cells. Although type II diabetes is on the increase each year, possibly due to bad eating habits of modern society, research on the culprit for this disease is still in its early days. In addition, unlike the culprit for Alzheimer's disease, amyloid *β*-peptide, amylin has failed to receive attention worthy of being featured in an abundance of review articles. Thus, the aim of this paper is to shine the spotlight on amylin in an attempt to put it onto the top of researchers' to-do list since the secondary complications of type II diabetes have far-reaching and severe consequences on public health both in developing and fully developed countries alike. This paper will cover characteristics of the amylin aggregates, mechanisms of toxicity, and a particular focus on inhibitors of toxicity and techniques used to assess these inhibitors.

## 1. Introduction

As mused by Reynaud, “as our life expectancy increases, the chances of getting a degenerative disease also increases…caused by something conceptually quite simple…incorrect protein folding” [1]. There are currently more than a dozen protein misfolding diseases which have been classified as amyloid diseases, with an equivalent number of amyloidogenic proteins responsible for each of them [2]. The amyloid diseases include Alzheimer's disease [3–9], Parkinson's disease [10, 11], Huntington's disease [11], Prion disease [12], primary and secondary systemic amyloidosis [2], and type II diabetes [13–18] for which the responsible misfolded protein is amyloid *beta* (A**β**), huntingtin, **α**-synuclein, prion protein (PrP), immunoglobulin (Ig) light chain, serum amyloid A, and amylin, respectively. Other than Alzheimer's disease, type II diabetes is the most prevalent in modern society with currently 346 million diabetic people world-wide, and the World Health Organization (WHO) predicts that the number of deaths that result from this disease will double between 2005 and 2030 [19]. Type II diabetes is responsible for a number of secondary complications such as heart attack, stroke, blindness, and renal failure [20], and thus research on amylin is of paramount importance in the fight against this debilitating disease.

**Figure 1:**
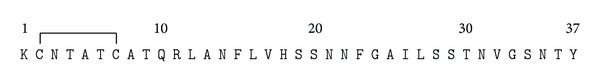
Amino acid sequence of amylin. Redrawn from Cooper et al. [21].

Amylin, also referred to as islet amyloid polypeptide (IAPP), is composed of 37 amino acid residues and as seen in Figure 1 contains a disulfide bridge between residues two and seven.

Amylin is derived after an 89-amino acid long precursor protein, referred to as preProIAPP, which is cleaved at the *N*-terminal yielding ProIAPP and which is subsequently posttranslationally processed by the prohormone convertase (PC2) [22]. These processes occur in pancreatic **β** cells, and, hence, amylin is secreted together with insulin in a 20 to 1 molar ratio of insulin to amylin [23]. Insulin is released following a diet rich in carbohydrates, as it is the hormone responsible for lowering blood glucose levels. Insulin functions by the following strategies: stimulation of mainly the muscle cells and adipocytes to increase their uptake of glucose, activation of enzymes responsible for glucose metabolism, increasing conversion of glucose to glycogen, inhibition of protein and lipid breakdown, and stimulation of their synthesis [20]. Initially, it was reported that amylin works antagonistically to insulin by inhibiting glycogenesis and promoting glycolysis [24–27]. However, other studies have suggested that amylin plays a critical role in glucose homeostasis by suppressing the release of glucagon from pancreatic **α** cells and, hence, prevents release of glucose from the liver, decreases gastric emptying, and stimulates the satiety center in the brain [23, 28–31]. The latter two events are important features that prevent an individual from feeling hungry thereby averting the condition of having even more stored glucose being released into the blood. Since amylin is coreleased with insulin, consuming an excess amount of carbohydrates and fat may lead to an elevated amount of amylin being secreted that could eventually initiate amylin aggregation, since it was found that a high carbohydrate or high fat diet promoted amyloid formation in transgenic mice [32, 33].

Amylin aggregation has been suggested to occur in a stepwise manner, with soluble monomeric amylin forming oligomeric structures, protofibrils, and eventually amyloid fibrils, some of which are toxic to the pancreatic *beta* cells [34]. Destruction of the pancreatic *beta* cells results in decreased insulin production and manifests as type II diabetes, a condition that is characterized by excess extracellular glucose with an intracellular deficit. The most commonly used treatment for diabetes is metformin and insulin. Although these two therapeutic agents help to manage the disease, they do not stop progression nor do they cure this debilitating disease.

An extensive review on amylin was conducted eleven years ago by Kapurniotu which focused primarily on the morphological and structural features of amylin aggregates, mechanism of aggregation, and the effects of amylin on cell viability with a brief overview on the potential of *N*-methylated peptides as inhibitors of amylin-mediated cytotoxicity [13]. More recently, two extensive reports on amylin have been published. One of the review articles focuses on the structural characteristics of amylin, mechanisms of its aggregation, factors affecting aggregation, and touched briefly on mechanisms of toxicity and inhibitors of aggregation [17], whilst the other gave a very insightful overview of the physiological and pathophysiological role of amylin and the toxic species of amylin and briefly highlighted a potential mechanism of amylin pathogenesis [18]. 

This paper aims to provide an in-depth and up-to-date overview of the molecular mechanism of amylin aggregation, all possible mechanisms of amylin-mediated toxicity, techniques used to evaluate inhibitors of fibril formation and toxicity, compounds that have been tested as potential inhibitors of amylin-mediated cytotoxicity, as well as a brief overview of the chemical strategies that are used to synthesize amylin.

## 2. Molecular Mechanism of Amylin Aggregation

Since amylin aggregation is central to this peptide acquiring cytotoxic properties, numerous researchers have over the last two decades either observed or proposed the molecular mechanism and region responsible for amylin aggregation (Table 1), with a common feature among all studies being that the aggregates were in a **β**-sheet conformation. The first study on the amyloidogenic region of amylin was performed by Westermark et al. and based on the nonamyloidogenic nature of amylin from different species and experimental data using synthetic peptides proposed that the 25–29 region is the shortest amyloidogenic region of amylin [35]. With the exception of the 1–7 region of amylin, the entire length of this peptide has at some stage been shown to have amyloidogenic properties (Table 1). Of note, it was found that the 22–27 region coiled around each other into typical amyloid fibrils [36] and also increased fibril formation [37]. In addition, the 11–20 region was found to bind to amylin with the highest affinity when compared to peptides that were homologous to other regions of amylin and that the 14–18 region is the core recognition site for amylin binding [38].

**Figure 2:**
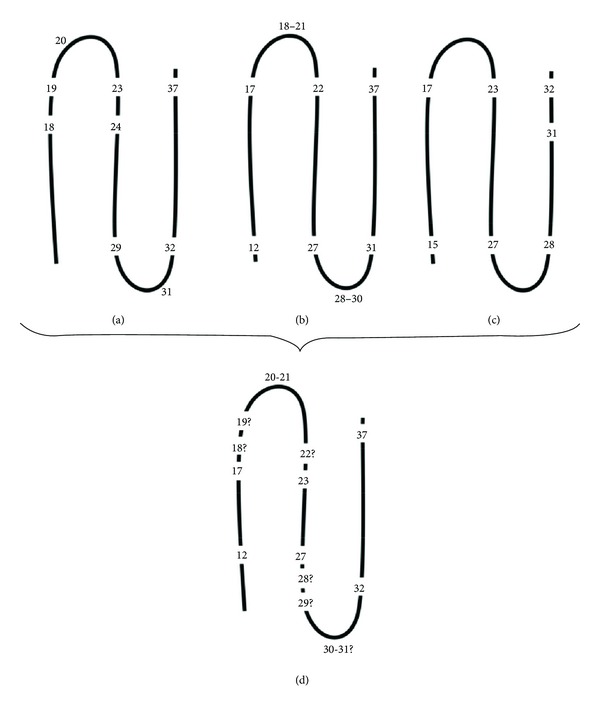
Schematic representation of the **β**-sheet and **β**-turn regions of amylin as predicted by (a) Jaikaran et al., (b) Kajava et al., and (c) Luca et al. [39–41]. Data integration for a comprehensive understanding of previous predictions ((a), (b), and (c)) is illustrated in (d).

One of the earliest models for **β**-sheet formation was proposed by Jaikaran et al. (Figure 2(a)) [39]. According to this model, a **β**-turn is predicted at residues 31 thereby allowing the 24–29 and 32–37 regions to form an antiparallel **β**-sheet and at residue 20 which would allow the 18–23 region to extend the **β**-sheet [39]. This model also proposes that hydrophobic interactions are responsible for initiating the aggregation process and that hydrogen bonds stabilize the **β**-sheet structure [39]. A later study proposed that the 12–17, 22–27, and 31–37 regions form antiparallel **β**-sheets with the 18–21 and 28–30 regions forming the **β**-turns (Figure 2(b)) [40]. In addition, it was also suggested that the hydrophobic side chains in the 15–17 and 32 region interact with that of the 23–27 region whilst there is interstrand hydrophilic association between the 28–31 regions of amylin strands (Figure 2(c)) [41]. It is noteworthy that there is considerable overlap between the **β**-sheet forming regions proposed by all three model-predicting studies. In addition, the predicted **β**-sheet forming regions contain the proposed nucleation sites for aggregation [48] as well as aromatic amino acids which have been reported to play a significant role in amyloid formation due to interactions between the planar aromatic structures which are also referred to as **π**-**π** interactions [49]. Taking all models into consideration, a proposed model of the **β**-sheet and **β**-turn regions of amylin is illustrated in Figure 2(d). This model proposes that the 12–17, 23–27, and 32–37 regions make up the **β**-sheet structure with regions 20-21 being constituents of the **β**-turn region. Residues 18, 19, 22, and 28–31 could either participate in forming the **β**-sheet or **β**-turn.

**Figure 3:**
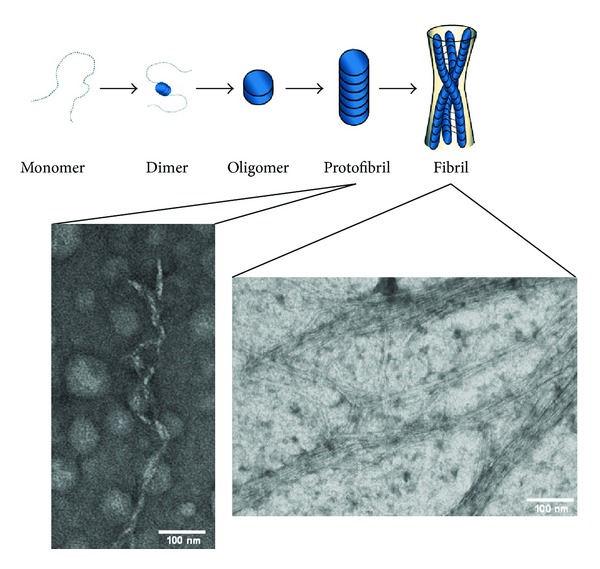
Schematic process of amylin forming nanoparticulate fibrils. Adapted from Dobson [50].

Thus, soluble monomeric amylin can associate into soluble **β**-sheet oligomeric state [51] which further progresses to protofibrils and insoluble amyloid fibrils [52]. According to Kodali and Wetzel, the oligomer which is formed prior to the protofibril is defined as being a “…metastable multimer in an amyloid formation reaction” [34]. These soluble intermediates were reported to have diameters between 2.7 and 4 nm, whilst protofibrils have a width of 5 nm and are “…non-spherical filamentous structures lacking a periodic substructure that are often found at intermediate times during the formation of mature fibrils,” and amyloid fibrils, are “…relatively straight, unbranched protein fibrils, with diameters in the 10 nm range, and often (but not always) consist of multiple protofilaments twisted around the fibril axis” (Figure 3) [34, 53]. Analysis of amyloid fibrils has revealed that individual **β**-strands are orientated perpendicular to the long fiber axis and thus form **β**-sheets [54–57]. Research studies by Goldsbury et al. revealed that amylin fibrils grow at both ends at a rate of approximately 1.1 nm per minute and that the width of fibrils ranged between 6 and 8 nm [53]. Thus, there is still no conclusively accepted size of each of the different types of amylin aggregates. 

## 3. Mechanism of Toxicity

Previously there was general acceptance that the fibrillar form of amylin is the toxic species [58–66]; however, the more recent consensus is that the soluble oligomeric structures exert the toxic effect [67–73]. Two noteworthy experiments for the latter hypothesis were conducted in 2006 and 2010 [73, 74]. Meier et al. evaluated rifampicin as a potential inhibitor of type II diabetes and found that although it did prevent fibril formation, and toxicity of amylin was still present, thus concluding that the soluble oligomers are the toxic species [74]. The second study showed that the fibrillar species of amylin was positively correlated with longevity of transgenic mice, once again suggesting that the prefibrillar or oligomeric form is toxic [73]. Although there is currently a general consensus that the oligomeric form of amylin is the toxic species, there are numerous theories regarding its mechanism of cytotoxicity. 

The first mechanism of toxicity postulated is membrane disruption and subsequent disturbance of intracellular homeostasis. It was initially reported by Westermark et al. that amylin disrupts cell membranes thereby causing cell death [35]. Thereafter, Lorenzo et al. exposed islet cells sandwiched between coverslips as well as unprotected cells to human amylin aggregates and found using Nomarsky microscopy that amylin interaction with cell membranes was crucial for toxicity [75]. Subsequent studies supported this theory by demonstrating that amylin aggregates formed pores or channels in lipid bilayers [59, 68, 69, 76]. Planar phospholipid bilayer membranes were used to demonstrate that nonselective ion voltage-dependent channels were formed in the presence of amylin [59]. This will promote the influx of Ca^2+^ and Na^+^ and K^+^ efflux and thereby disrupt ionic homeostasis [59]. Kayed et al. also employed lipid bilayers and showed that there was increased conductance in the presence of amylin oligomers and fibrils [69]. In addition, intracellular calcium levels were found to be elevated after exposure to amylin, and it is noteworthy that destabilization of intracellular Ca^2+^ homeostasis was a mechanism used by other amyloidogenic peptides to induce toxicity [58, 77]. 

Subsequent studies also demonstrated that fibril formation was increased in the presence of anionic lipid membranes [56, 78], and it was suggested that electrostatic interactions between amylin and the negatively charged lipids on membranes are responsible for amylin association with the cell membrane [79]. In addition, amylin was found to insert into membranes and incorporate membrane lipids into the growing amyloid fibril, thereby causing membrane disruption [79–82]. Using human islets and amylin oligomers, Ritzel et al. demonstrated that amylin oligomers could promote the loss of rat pancreatic **β**-cells (RINm5F) by disrupting cell membranes as well as disruption of islet architecture such as cell-to-cell adherence, both of which are crucial for cell survival [72]. For more details on amylin-mediated membrane disruption, see recent reviews by Engel and coworkers [16, 83]. It thus seems that membrane disruption could be a leading cause of amylin-mediated toxicity.

The second proposed mechanism of amylin-mediated toxicity is generation of reactive oxygen species (ROS) such as hydrogen peroxide (H_2_O_2_), which results in cell death [58]. It was also shown that generation of ROS was a mechanism used by other amyloidogenic peptides for toxicity [58]. At the same time, Schubert et al. detected peroxides using 2′,7′-dichlorofluorescein diacetate, and they also demonstrated that amylin increased the accumulation of H_2_O_2_ in B12 cells [84]. 

The third hypothesis of amylin-mediated toxicity is apoptosis. Apoptosis is defined as programmed cell death and is characterised by cell shrinkage, membrane blebbing (detachment of the cell membrane from the cytoskeleton), disruption of nuclear architecture, and DNA laddering (breaking of chromosomal DNA into fragments containing 180 base pairs) [85].

In early experiments, Lorenzo et al. showed that aurintricarboxylic acid, an endonuclease inhibitor that stops apoptosis, is able to reduce amylin-mediated toxicity of islet cells [75]. They had also stained islet cells with propidium iodide and using epifluorescence microscopy showed that there was chromatin condensation and using agarose gel electrophoresis revealed that DNA fragmentation had also occurred [75]. 

**Figure 4:**
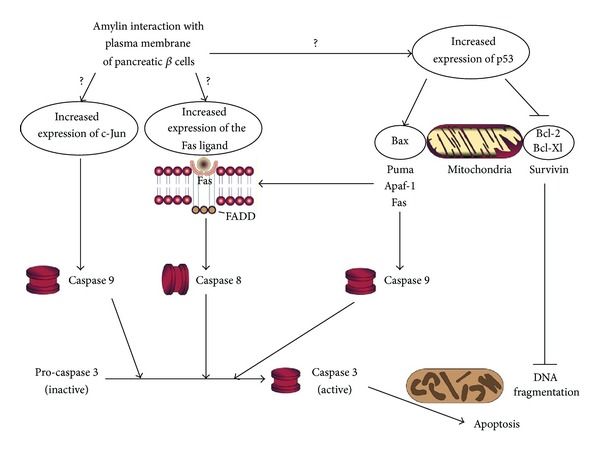
The proposed roles of the c-Jun, Fas, and p53 proteins in apoptosis induced by human amylin in pancreatic *beta-*cells.

Thereafter, the TUNEL assay and gel electrophoresis was employed to reveal that amylin-induced DNA fragmentation and apoptosis in RINm5F cells [86]. This research team also used quantitative Northern blot analysis to demonstrate that amylin increased expression of the p21 and p53 tumor suppressor genes, both of which encode for proteins that arrest cell proliferation, leading to apoptosis (Figure 4) [86]. This finding was later supported by transmission electron microscopy and scanning electron microscopy analysis of RINm5F cells that were exposed to amylin which clearly showed ultrastructural evidence of apoptotic damage [87]. The theory that apoptosis is the mechanism by which amylin causes cell death was further supported by the finding that amylin increases the expression of c-Jun, a gene that is involved in the apoptotic pathway (Figure 4), in RINm5F and the human insulinoma cell line (CM) [88]. Huang et al. later showed that amylin could trigger endoplasmic reticulum stress-induced apoptosis [89]. In the presence of amylin, levels of Fas/Fas ligand (FasL) and Fas-associated death domain (FADD), both of which are involved in apoptosis, were elevated (Figure 4) [90]. More recently, it was concluded that amylin oligomers induced elevated cytosolic levels of Ca^2+^ in the rat insulinoma cell line INS 832/13 that resulted in hyperactivation of the protease calpain-2, leading to apoptosis [91].

The previously mentioned mechanisms could possibly work together to eventually result in cell death. For example, membrane damage caused by the toxic species of amylin or ROS damage could potentially result in activation of the apoptotic pathway, eventually resulting in cell death. Previous studies have also shown that there is significant overlap of the different mechanisms responsible for amylin-mediated cytotoxicity. One such study used rat cortical neurons and demonstrated that amylin aggregates induced the apoptotic genes c-Jun, junB, c-fos, and fosB, as well as the oxidative stress genes cox-2 and l*κ*B-**α** [92]. In addition, oligomers were found to contribute to membrane instability of voltage-clamped planar bilayer membranes by increasing conductance and electrical noise in the membrane as well as inducing the formation of abnormal vesicle-like membrane structures which resulted in apoptosis [67]. Gurlo et al. performed *in vivo* experiments with an oligomer-specific antibody and cryoimmunogold labeling and showed that the toxic oligomer is present in the secretory pathway and is able to disrupt membranes herein as well as mitochondrial membranes [93]. These events result in cellular dysfunction and apoptosis [93]. Lim et al. further supported the hypothesis that the mechanism of amylin toxicity is membrane disruption by showing that mitochondrial proteins were deregulated when SH-SY5Y neuroblastoma cells were exposed to amylin [94]. However, this group also showed that production of ROS increased when cells were exposed to amylin [94]. 

Taking into account all of the proposed and observed mechanisms of amylin-induced cytotoxicity, it thus appears that membrane disruption, generation of ROS, and apoptosis are interrelated. Membrane disruption appears to have a direct effect on apoptosis, and thus these two mechanisms could actually be working together to induce amylin-mediated cytotoxicity.

## 4. Techniques Used to Monitor Aggregation and Toxicity

There are numerous techniques that are employed to monitor amylin aggregation and toxicity, and inhibition thereof.

**Figure 5:**
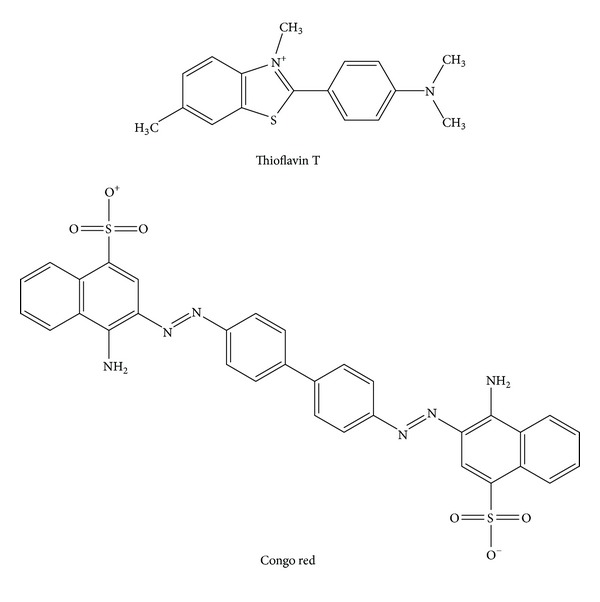
Structures of Thioflavin T and Congo red. Adapted from Aitken et al. [95].

Two commonly used dyes for identification of the fibrillar form of amylin are Congo red and thioflavin T (ThT) (Figure 5). It was found that when bound to amyloid fibrils, Congo red produced a characteristic yellow-green birefringence under polarized light, and it was assumed that this dye interacted with the **β**-sheet structure that is present in all amyloids [96, 97]. Thus, Congo red staining was initially used for classification purposes to ascertain if an aggregate is amyloidogenic in nature [21, 96, 97]. Since Congo red staining is relatively easy to perform, it has also been used to identify amyloid fibrils in pancreatic islets as well as in cell-based assays [80, 96, 98]. In addition, Congo red staining was used to identify the shortest fragment of amylin that retained amyloidogenic properties [36], to assess if modifications of the amylin structure could alter its amyloidogenic potential [61, 99], and to evaluate potential inhibitors of amylin fibril formation [64]. However, Congo red staining is neither very sensitive nor specific as it has been shown to stain amorphous aggregates [98] that can bind to cellular membranes [100]. Moreover, since Congo red is thought to bind **β**-sheet structures, it is quite possible that nonspecific interactions could occur in a cell-based system, components of which may have **β**-sheet structures.

The other molecule used to detect amyloid fibrils is thioflavin T (ThT) [101]. ThT is a thiazine dye that has a maximal excitation at 385 nm and emission at 445 nm. When attached to amyloid fibrils, however, the maximum excitation is 450 nm and the emission is enhanced at 485 nm [101]. The ThT assay is thus one of the most widespread assays used to follow amylin aggregation by being able to detect the fibrillar species of amylin [69, 102] and also to screen molecules as potential inhibitors of amylin fibril formation [60, 65, 74, 90, 95, 103, 104]. However, to date, the exact mechanism of ThT binding to amyloid fibrils is yet to be identified. One hypothesis draws on the fact that ThT has both polar (the benzothiazole group containing nitrogen and sulphur) and hydrophobic (the dimethylamino group attached to a phenyl group) regions thereby allowing micelle formation in an aqueous environment [105]. The positively charged nitrogen pointing outside could then hydrogen bond to hydroxyl groups on amyloid fibrils, causing a change in excitation leading to enhanced fluorescence emission [105]. Although the ThT assay is very simple to perform, it does not yield quantitative data and could produce false positive results in the presence of amorphous aggregates.

Although Congo red and ThT can detect amylin aggregates, they do not indicate if these aggregates are the oligomeric or fibrillar species.

To this end, microscopy techniques increased in popularity, and transmission electron microscopy (TEM) became the technique of choice for visualizing amylin fibrils. Similar to the previous two techniques, TEM was used to identify the shortest fragment of amylin that formed typical amyloid fibrils [36, 45], to assess if modifications of the amylin sequence could alter its amyloidogenic potential [22, 61, 99], to follow amylin aggregation [68, 69, 102], and, hence, to assess the potential of molecules as inhibitors of amylin fibril formation [37, 60, 64, 65, 95, 104, 106]. Although it is relatively easy to prepare the sample grids for TEM, image analysis requires a certain degree of skill and can be quite time-consuming with each grid taking hours to be visually assessed. Since a very small volume of sample is used on each grid, fibrils can be missed leading to false negative results. However, scanning transmission electron microscopy (STEM) made a significant contribution to understanding the conformational changes during amylin agrgegation as it was the first technique used to determine the size of amylin aggregates that form [43, 107]. Some of the ground-breaking data garnered from STEM are that the amylin protofibril is 5 nm in width, higher order fibrils are formed by coiling of two protofibrils with a 25 nm axial cross-over repeat and are 8 nm in width, and that each 1 nm of protofibril length contains 2.6 human amylin molecules [107]. In addition, STEM-generated data were used in part to predict previously described models for **β**-sheet formation [40, 41].

The other technique that allows visualization of amylin fibrils is atomic force microscopy (AFM). This technique was used to visualize fibril formation from full length amylin as well as a fragment of amylin that is homologous to the 20–29 region and to size the aggregates that formed [53, 108]. One of the significant findings of AFM-based studies on amylin is that fibrils grow at a rate of 1.1 nm/minute and that the growth of the fibril is bidirectional [53]. The drawback to this technique is that it is not quantitative, but it is labour-intensive, and samples must be adsorbed to a mica surface in order to monitor fibril growth over time. The latter drawback is a cause for concern since it has been reported that the type of amylin fibrils that form in the presence of a mica surface is significantly different in morphology from that formed free in solution [53, 108]. Moreover, the atomic force microscope is a very expensive instrument and extensive training is required for the implementation of this technology. 

Since amylin aggregates adopt a **β**-sheet structure, techniques such as circular dichroism (CD) and Fourier transform infrared spectroscopy (FT-IR) that are able to give insight into the secondary structure of the peptide were extensively used. Circular dichroism (CD) is based on the concept that random coil structures have a maximum absorbance at 220 nm and minimum at 200 nm, and the **α**-helix state absorbs maximally between 190 and 195 nm with a minimum absorbance between 208 and 222 nm, whilst **β**-sheets have a maximum absorbance between 195 and 200 nm and a minimum between 215 and 220 nm [37, 61]. FT-IR spectroscopy is similar to CD in that it also uses differences in absorbance to identify the secondary structure of amylin [13, 35, 63, 99]. A maximum absorbance at 1625–1630 cm^−1^ indicates the presence of **β**-sheets, whereas a maximum absorbance at 1660–1670 cm^−1^ reveals that a random coil structure is present [13]. These peaks are due to the stretching vibration of C=O and C–N groups, and the shift to lower values is an indication of decreased hydrogen bonding interactions between these groups.

Both CD and FT-IR spectroscopy techniques have been employed to determine the amyloidogenic region of amylin, the minimal sequence of amylin that retained fibrillogenic properties [36, 38, 39, 42, 45], and to monitor amylin aggregation and, hence, to elucidate the effect of potential inhibitors on the secondary structure of amylin [13, 22, 37, 61, 64, 65, 68, 99, 102, 103, 106, 109]. Interestingly, CD was one of the earliest techniques used to determine that the oligomeric form of amylin is in a **β**-sheet conformation [109]. Although CD and FT-IR spectroscopy techniques are easy to perform and can accurately determine the secondary structure of amylin, these techniques suffer a major drawback when used as a screening technique for potential inhibitors of amylin-induced cytotoxicity. This is due to the observation that both the oligomeric and fibrillar forms of amylin are in a **β**-sheet conformation, and thus these techniques cannot differentiate between these two species of amylin [52, 109]. Since it is widely accepted that the oligomeric form of amylin is cytotoxic whilst the fibrillar form is nontoxic [67–73], neither CD nor FT-IR spectroscopy techniques can be used exclusively to screen inhibitors of amylin-mediated cytotoxicity. 

The sedimentation/precipitation assay is another technique that has been used to determine the effect of peptides and compounds on amylin fibril formation [37, 95, 103]. Initially, this technique made use of the intrinsic fluorescence of tyrosine [37]. At specific time points, peptide samples are centrifuged and emission spectra of the supernatant are documented to determine the amount of amylin that has not precipitated and thus the amount of amylin that has aggregated can be ascertained [37]. An improved version of this assay made use of trace amounts of radio-labeled amylin, which are added to native amylin in the presence or absence of potential inhibitors [95]. After centrifugation, the amount of radio-labeled amylin remaining in the supernatant is determined to give an indication of the amount of amylin that has been used for fibril formation. Although this assay cannot differentiate between fibrils and oligomers, it has been used in conjunction with other assays to evaluate the potential of compounds as inhibitors of amylin-induced cytotoxicity [37, 95, 103].

Thus, except for AFM, all previously mentioned techniques cannot solely identify amylin oligomers, and since the oligomer is the toxic species [67–73], these techniques cannot be used to evaluate compounds as potential inhibitors of amylin-mediated cytotoxicity.

The technique that clearly identifies the suitability of a compound as a potential therapeutic agent for type II diabetes is the cytotoxicity assay. This assay is conducted by exposing mammalian cells to amylin alone or mixtures of amylin and potential inhibitors and evaluating cell viability after a set period of time. The following cell lines have been used for assessing inhibition of amylin-mediated cytotoxicity, PC12 (rat phaeochromocytoma cells), HIT-T5 (Syrian Hamster *beta* cells), HTB-14 (human glioblastoma/astrocytoma cells), and RIN-5F (rat pancreatic *beta* cells) [22, 36, 37, 60–62, 64, 65, 86, 87, 95, 103, 106, 110]. The RIN-5F cell line is most commonly utilized to date for testing inhibitors of amylin-mediated toxicity possibly due to the fact that it is a pancreatic *beta* cell line and, hence, the target of amylin-mediated cytotoxicity as would occur in an *in vivo* system.

The terminal deoxynucleotidyl transferase-mediated dUTP nick-end labeling (TUNEL) [71, 72, 89, 111], alamar blue [37, 110, 111], caspase [90], and (3-[4,5-dimethylthiazol-2-yl]-2,5-diphenyl tetrazolium bromide (MTT) [22, 36, 62, 64, 71, 103, 106] assays have been used to assess cytotoxicity, with the TUNEL and caspase assays detecting apoptosis specifically. 

The MTT assay is, however, the most favored and relies on the fact that mitochondrial dehydrogenase that is present in actively metabolizing cells is able to cleave the tetrazolium salt 3-(4, 5-dimethylthiazol-2-yl)-2, 5-diphenyltetrazolium bromide (MTT) to yield purple formazan crystals. These crystals are thereafter solubilized and spectrophotometrically analyzed to yield quantitative data with respect to cell viability as the amount of purple crystals formed is directly proportional to the amount of viable cells present [112]. An improvement on the MTT assay is the MTS assay which involves a single-step protocol with the formazan product readily dissolving in cell culture medium thus reducing the assay time [113]. Although the MTT and MTS assay gives quantitative data with regard to the protective function of compounds, it is very expensive and time-consuming as it is dependent on the growth rate of a particular cell line.

To the best of our knowledge, the only other cell-based technique for monitoring inhibition of amylin-mediated cytotoxicity makes use of fluorescence microscopy. This technique involves use of fluorescent-labeled amylin (Bodipy-amylin) and a fluorescent cellular membrane marker (Texas Red-DHPE), as well as cell imaging using a confocal microscope [100]. Once cells are exposed to amylin and the test compound, this technique allows detection of any changes in cell morphology that would indicate cell death. One of the most defining features of amylin-mediated cytotoxicity is the loss of cellular membrane integrity, and this can be easily visualized using fluorescent-labeled amylin and a fluorescent cell membrane marker. Although this type of investigation gives excellent qualitative data, it has certain drawbacks. As with the cytotoxicity assay discussed earlier, this technique requires growth of mammalian cell lines which is both expensive and time-consuming. It is also critical to ensure that the fluorescent label does not interfere with the aggregation kinetics and toxic properties of native amylin. It is therefore not feasible to use fluorescence microscopy as a screening technique for inhibitors of amylin-mediated toxicity.

Seeing as cell-based systems can be quite time-consuming and labour intensive, and that no cell-free technique can be used exclusively to detect the oligomeric form of amylin, it is quite evident that a breakthrough is needed in development of a technique that would allow efficient screening of potential inhibitors of amylin-mediated cytotoxicity.

## 5. Inhibitors of Amylin-Mediated Cytotoxicity

The initial strategy to design inhibitors of amylin-mediated cytotoxicity was based on the hypothesis that generation of ROS is the mechanism of toxicity. ROS is able to damage DNA and oxidizes the constituent amino acids of proteins as well as polyunsaturated fatty acids that are present in lipids, all of which can lead to apoptosis. ROS damages DNA by causing strand breakage, base modification, oxidation of deoxyribose, and DNA-protein cross-links [114]. Since cell membranes are made up of a large amount of polyunsaturated fatty acids, oxidation by ROS causes detrimental changes in membrane fluidity, permeability, and metabolic functions. Oxidative damage to proteins results in either protein degradation or alteration of its properties such as causing a soluble protein to aggregate [114].

Thus, to circumvent the toxic effect of amylin-generated ROS, a number of quinone derivatives that were known to scavenge free radicals were evaluated [60]. However, it was found that only rifampicin and its analogues *p*-benzoquinone and hydroquinone inhibited the toxic effect of amylin whereas other antioxidants with scavenging ability did not exhibit any inhibitory effect on amylin toxicity [60]. This study was the first to observe that an inhibitor (rifampicin) could bind to amylin aggregates and prevent its attachment to the cell surface [60]. It was thus suggested that rifampicin and its analogues could have a dual mechanism to exert their protective function, preventing amylin-cell interaction and also scavenging ROS [60]. The thiol antioxidants *N*-acetyl-L-cysteine, the reduced form of glutathione, and dithiothreitol were also found to significantly decrease amylin-mediated apoptosis whereas the free radical scavengers catalase and *n*-propyl gallate did not [117]. These results indicated that a protective function can be achieved by inhibiting the signalling pathways that are regulated by the redox state of thiol-containing molecules as these could be responsible for amylin-induced cytotoxicity [117]. 

Thus, although amylin-mediated toxicity involves generation of ROS, as stated previously, the toxic oligomeric species of amylin facilitates membrane disruption and could trigger a number of events that lead to apoptosis and cell death [67, 93]. To this end a significant amount of research focused on the core problem, inhibiting formation of the toxic species of amylin.

**Figure 6:**
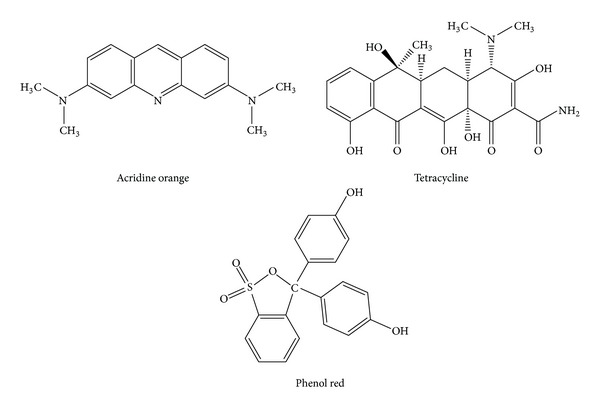
Structures of acridine orange, tetracycline, and phenol red. Adapted from Aitken et al. and Gazit [95, 115].

Based on the finding that heteroaromatic interactions between amylin and polycyclic compounds resulted in geometric constraints that reduced fibrillogenesis, Aitken et al. highlighted the potential of polycyclic compounds as inhibitors of amylin-mediated cytotoxicity by reporting that Congo red, acridine orange, and tetracycline could reduce amylin fibrillogenesis [95]. However, cytotoxicity testing was only performed with Congo red as it had no intrinsic toxicity. Two separate studies found that Congo red reduced amyloid formation and ROS production and subsequently decreased *beta* cell apoptosis [95, 118]. Another polycyclic compound, phenol red (Figure 6), was also found to reduce amylin-mediated cytotoxicity on *beta* cells, once again highlighting the role that heteroaromatic interactions have in inhibiting formation of amylin fibrils [106]. 

The polyphenolic compound resveratrol that is found in grapes and red wine was also shown to significantly inhibit fibril formation of amylin and the associated cytotoxicity [119], and based on replica-exchange molecular-dynamics simulations, it was proposed that resveratrol reduces fibrillogenesis by preventing lateral growth of the amylin **β**-sheet [120]. A recent review indicated that even though resveratrol could have an impact on diabetes by a multitude of mechanisms as evidence by *in vitro* testing, few clinical human trials have been conducted possibly due to its poor bioavailability [121]. To date, there is only one peer-reviewed human clinical trial that monitored the effect of resveratrol on insulin sensitivity in type II diabetes [122]. Although it was found that resveratrol does reduce ROS [122], its effect on amyloid formation or inhibition of amylin-mediated cytotoxicity is yet to be monitored in an *in vivo* system.

Interestingly, the only study that has made use of an *in vivo* system to evaluate the effect of a known antiamyloidogenic agent on diabetes was performed by Aitken et al. [73] and Forloni et al. [123]. This study made use of transgenic mice and demonstrated that tetracycline could delay onset of diabetes and could also delay the progression of this disease [73]. However, to truly probe whether tetracycline acts by inhibiting amyloid formation, histopathological analysis would have been necessary and it is unfortunate that this type of examination was not performed.

Extending the search for molecules that could prevent amylin aggregation and its subsequent cytotoxicity, attention turned to peptides since it has low toxicity and high specificity and thus could be a viable option as a therapeutic agent. Since analysis of the rat amylin sequence implied that the unique presence of proline residues could be responsible for the lack of amyloid formation in rodents, the design of an inhibitor containing a proline substitution was encouraged. With this in mind, Abedini et al. synthesized full length amylin but substituted the serine at position 26 with proline and found that this modified peptide could bind to amylin and prevented fibril formation [124]. A possible explanation for this observation is that proline is known to induce **β**-turns in peptides [125]. Fibril growth requires **β**-sheet conformation of incoming amylin chains, and a modified bent peptide binding to amylin will therefore disrupt the free stacking of **β**-sheet amylin molecules. Although this modified form of amylin inhibited amylin-mediated cytotoxicity, another amylin derivative that contains three substitutions with proline at residues 25, 28, and 29 was already undergoing clinical trials [126–139]. This amylin derivative was initially called symlin and thereafter marketed as pramlintide and is used as an adjunct to insulin in the management of type II diabetes [126–139]. However, it should be noted that this peptide has not been evaluated as an inhibitor of amylin aggregation or amylin-mediated cytotoxicity. 

However, two other therapeutic agents of diabetes, Metformin and Rosiglitazone, were evaluated in an *in vivo* system to determine their effect on amyloid formation [140]. One of the therapeutic functions of Metformin and Rosiglitazone is to increase insulin sensitivity and, hence, reduce secretion of insulin from pancreatic *beta* cells [140]. In this study, transgenic mice that express amylin were treated with either Metformin or Rosiglitazone for twelve months, and subsequent histopathological analysis revealed that these therapeutic agents significantly reduced the amount of amyloid deposits that formed in the pancreata of the treated animals [140]. As previously mentioned, amylin is secreted together with insulin from pancreatic *beta* cells, and thus it was suggested that both Metformin and Rosiglitazone could possibly reduce amyloid formation by decreasing the amount of amylin secreted [23, 140]. This study thus highlights the need for developing molecules that could either prevent amylin secretion or reduce amyloid formation from amylin.

Since full length amylin is difficult to chemically synthesize, attention shifted to shorter peptide sequences as potential inhibitors of amylin aggregation and subsequent amylin-mediated cytotoxicity. A breakthrough was made when Rijkers and coworkers found that the introduction of *N*-alkylated amino acids or ester functionalities into peptide sequences allowed the peptides to behave as **β**-sheet inhibitors that prevented the formation of toxic amylin **β**-sheets [116].

**Figure 7:**
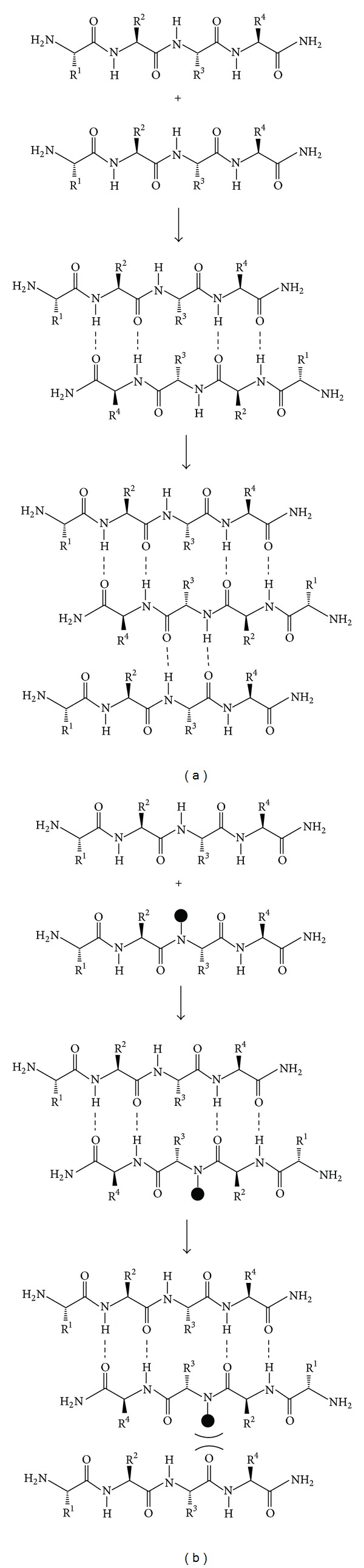
Diagram showing how (a) unmodified amino acids can form **β**-sheet structures via hydrogen bonding (represented as - - -) and how (b) *N*-methylation (expressed as dark circles) replaces hydrogen in a polypeptide and thus preventing **β**-sheet stacking. R^1^–R^4^ represent the side groups of the amino acids. Adapted from Rijkers et al. [116].

As depicted in Figure 7(a), single amylin strands form **β**-sheets by hydrogen bonding between *N*–*H* and *C*=*O* dipoles that point outward from the amylin backbone. However, peptides containing *N*-alkylated amino acids and which exhibit a **β**-sheet conformation are able to bind to native amylin and prevent attachment of any further peptide strands by disrupting the hydrogen-bonding capacity of the peptide and by causing steric hindrance (Figure 7(b)). 

The presence of *N*-methylated amino acids improves the biostability of the peptide by being resistant to proteolysis and it also increases the membrane permeability of the peptide [141]. Employing this approach, numerous amylin derivatives that incorporated *N*-methylated amino acids were synthesized as potential inhibitors of fibril formation. The first of these inhibitors having the sequence SNNF(*N*-Me)GA(*N*-Me)ILSS (single letter notation of amino acids used and *N*-Me refers to *N*-methylated amino acids) was reported by Kapurniotu et al. in 2002 [64]. This amylin derivative was shown to inhibit aggregation of the 20–29 region of amylin and prevented its cytotoxicity [64]. It was also found that the presence of *N*-methylations allowed the peptide to exist in an ordered **β**-sheet structure which is of importance since a stable conformation is crucial if the peptide is to be used as an inhibitor [64]. However, it should be noted that the effect of this amylin derivative was not assessed using full length amylin. The amylin derivatives that were evaluated as potential inhibitors of cytotoxicity affected by full-length human amylin are presented in Table 2.

To the best of our knowledge, no *in vivo* testing was performed using any of the peptide inhibitors mentioned. It is, however, noteworthy that two patents have been granted for peptide derivatives of amylin that can prevent amylin aggregation and amylin-mediated cytotoxicity and which have been suggested as potential therapeutic agents of type II diabetes [143, 144]. However, the first patent was granted in 1996 whilst the second was granted in 2007, and to date, none of the derivatives mentioned has been used in clinical trials [143, 144]. Thus, there still exists the need for more potent peptide-based inhibitors of amylin-mediated cytotoxicity.

In addition to peptide derivatives of amylin, a diverse range of other molecules were more recently assessed as potential inhibitors of amylin aggregation and cytotoxicity. The membrane binding protein annexin A5 was shown to decrease the toxic effect of amylin on rat pancreatic **β**-cells by 90% [104]. Cabaleiro-Lago et al. showed that *N*-isopropylacrylamide:*N*-tert-butylacrylamide (NiPAM:BAM) copolymeric nanoparticles were able to decrease amylin aggregation [145]. They suggested that amylin could possibly be adsorbed onto the nanoparticle surface thereby decreasing the available amount in solution and thus increasing the lag time of fibrillation [145]. It was also found that sulfonated triphenyl methane derivative, acid fuchsin, and 3-(1-(4-amino-3-methyl-5-sulfonatophenyl)-1-(4-amino-3-sulfonatophenyl) methylene) cyclohexa-1,4-dienesulfonic acid decreased amylin-mediated toxicity by approximately 80% [146]. Rigacci et al. showed that oleuropein aglycon, the main phenolic component of extra virgin olive oil, reduced the toxicity of amylin by approximately 20% [147]. However, these molecules need to be scrutinized further to ascertain its biostability and more importantly whether it is biodegradable. It is noteworthy that the toxicity of nanoparticles has been reviewed twice with the conclusion being drawn that nanoparticles are not easily cleared from an *in vivo* system and thus could lead to toxicity [148, 149].

## 6. Amylin Synthesis

At an early stage, it was found that amyloidogenic proteins from different suppliers have different properties [150]. This was substantiated by a more recent study, which demonstrated the presence of impurities in commercially available proteins [151]. It was reported that the presence of impurities can affect the aggregation kinetics of amyloidogenic peptides [99, 152, 153], thus highlighting the need for synthetic strategies that yielded pure protein. To this end, Abedini and Raleigh published the first synthetic strategy for amylin [154]. They made use of pseudoproline derivatives, and to date there have been numerous improvements to their method [102, 155–158] with only one report that did not use pseudoproline derivatives [151]. It is therefore deemed critical that a more cost-effective and efficient synthetic strategy is required to generate amylin of a quality that would afford reproducible results.

## 7. Conclusion

It is thus quite evident that there exists a gap with respect to novel techniques that could enable fast and cheap screening of potential therapeutic agents for type II diabetes. That being said, although much progress has been made with respect to the type of inhibitor that could be used, peptide derivatives that could inhibit amylin-induced cytotoxicity could be developed further in a bid to find a potential therapeutic agent for type II diabetes. Most importantly, to gain more insight into amylin aggregation dynamics and also to screen potential inhibitors of amylin-mediated cytotoxicity, a cost-effective strategy to acquire sufficiently pure amylin is deemed critical.

## Figures and Tables

**Table 1:** Observed and predicted amyloid forming regions of amylin.

Year	Amyloidogenic region	Predicted or observed
1990 [35]	20–29	Observed
1999 [42]	17–34, 24–37, 30–37	Observed
2000 [43]	20–29	Observed
2000 [36]	23–27 and 22–27	Observed
2001 [44]	22–29	Observed
2001 [39]	8–20	Observed
2002 [38]	14–18, 14–22, 14–20, 15–20, 15–19	Observed
2002 [37]	22–27	Observed
2003 [45]	12–17, 15–20	Observed
2005 [40]	12–17, 22–27, 31–37	Observed
2006 [46]	13–18	Predicted
2007 [41]	8–17, 28–37	Predicted
2007 [47]	12–18, 15–20, 22–28	Predicted
2009 [48]	8, 13, 17, 25, 27, 32*	Predicted

*Nucleation sites.

**Table 2:** Amylin derivatives as potential inhibitors of cytotoxicty affected by full-length human amylin.

Amylin derivative	Position of *N*-methylated residue	Decrease in cytotoxicity	Cell line used	Reference
Amylin_20–25_		25%	RIN-1056A	[37]
Amylin_24–29_		0%	RIN-1056A	[37]
Amylin_12–17_		0%	RIN-1056A	[110]
Amylin_15–20_		0%	RIN-1056A	[110]
Amylin_22–27_	20 and 25	20%	RIN 5fm	[65]
Amylin_1–37_	24 and 26	50%	RIN 5fm	[103]
Amylin_13–18_		50%	RIN-1056A	[111]
Amylin_20–25_		50%	RIN-1056A	[111]
Amylin_3–6_		45%	RIN 5fm	[142]
Amylin_3–6_	3–5	0%	RIN 5fm	[142]
Amylin_9–13_		40%	RIN 5fm	[142]
Amylin_9–13_	9, 12, and 13	0%	RIN 5fm	[142]
Amylin_15–20_		20%	RIN 5fm	[142]
Amylin_15–20_	15–17 and 19	0%	RIN 5fm	[142]
Amylin_22–27_		40%	RIN 5fm	[142]
Amylin_22–27_	23–27	0%	RIN 5fm	[142]
Amylin_29–34_		50%	RIN 5fm	[142]
Amylin_29–34_	30 and 32–34	50%	RIN 5fm	[142]
